# Identification of HIV-1-specific regulatory T cells using HLA-class-II tetramers

**DOI:** 10.1186/1742-4690-9-S2-P279

**Published:** 2012-09-13

**Authors:** M Angin, PL Klarenbeek, M King, KW Wucherpfennig, MM Addo

**Affiliations:** 1 Ragon Institute of MGH, MIT and Harvard, Boston, MA, USA; 2 Brigham and Women's Hospital, Boston, MA, USA; 3 Dana-Farber Cancer Institute, Boston, MA, USA

## Background

Regulatory T cells (Tregs) are potent immune modulators whose role in HIV-1 immuno-pathogenesis remains inadequately understood. While “bulk” Treg populations have been studied extensively in the context of HIV-1 infection, no reliable data is available on HIV-1 specificity of Tregs and induction of these cells in infected individuals or vaccine recipients.

Part of the challenge in detecting antigen-specific Treg populations relates to the limited availability of visualization tools and the scarcity of the overall human Treg population -representing roughly 5% of CD4+ T cells with absolute Treg numbers further decreasing during HIV-1 disease progression.

## Methods

In order to screen for HIV-1 specific regulatory T cell populations, we first flow-sorted and expanded CD4+CD25+CD127low Tregs ex vivo from HLA DRB1*0401 expressing HIV-1 infected individuals. Expanded Tregs underwent analysis of function, phenotype, deep TCR sequencing and epigenetic analysis. Treg lines were then stained with HLA class II tetramers specific for HIV-p24-gag and appropriate controls.

## Results

Expanded Tregs were highly suppressive and displayed the phenotype of “activated” Tregs. Tregs were highly demethylated at the TSDR as shown by epigenetic analysis of the FOXP3 gene and showed an unskewed TCR repertoire compared to unexpanded ex vivo-sorted Tregs. Staining with HLA-class-II-tetramers specific for the HIV-p24-Gag epitope DRFYKTLRAEQASQ revealed a detectable response in one subject (an individual with chronic untreated progressive HIV-1 infection), at a frequency of 0.19% of CD4+ T cells in the non-enriched Treg culture. After tetramer-positive T cell enrichment, this frequency was increased to 6.14%.

## Conclusion

Our data represent the first identification of HIV-1-epitope-specific Tregs in HIV-1 infected individuals. Identification and further functional characterization of HIV-1 specific Tregs will provide important insight for the evaluation of vaccine strategies as these may lead to induction of not only HIV-1-specific effector populations but also HIV-1-specific regulatory T cells, which may negatively impact vaccine immunogenicity.

